# Environmental correlates of temporal variation in the prey species of Australian fur seals inferred from scat analysis

**DOI:** 10.1098/rsos.211723

**Published:** 2022-10-05

**Authors:** Kimberley Kliska, Rebecca R. McIntosh, Ian Jonsen, Fiona Hume, Peter Dann, Roger Kirkwood, Robert Harcourt

**Affiliations:** ^1^ School of Natural Sciences, Macquarie University, Sydney, Australia; ^2^ Research Department, Phillip Island Nature Parks, Victoria, Australia; ^3^ South Australian Research and Development Institute, South Australia, Australia

**Keywords:** El Niño–Southern Oscillation index, Southern Annular Mode, *Arctocephalus pusillus doriferus*, Bass Strait, *Sardinops sagax*, climate change

## Abstract

Marine ecosystems in southeastern Australia are responding rapidly to climate change. We monitored the diet of the Australian fur seal (*Arctocephalus pusillus doriferus*), a key marine predator, over 17 years (1998–2014) to examine temporal changes. Frequency of occurrence (FO) of prey was used as a proxy for ecosystem change. Hard part analysis identified 71 prey taxa, with eight dominant taxa in greater than 70% of samples and predominantly included benthic and small pelagic fish. FO changed over time, e.g. redbait (*Emmelichthys nitidus*) reduced after 2005 when jack mackerel (*Trachurus declivis*) increased, and pilchard (*Sardinops sajax*) increased after 2009. Using generalized additive models, correlations between FO and environmental variables were evident at both the local (e.g. wind, sea surface temperature (SST)) and regional (e.g. El Niño–Southern Oscillation Index (SOI), Southern Annular Mode (SAM)) scales, with redbait and pilchard showing the best model fits (greater than 75% deviance explained). Positive SAM was correlated to FO for both species, and wind and season were important for redbait, while SOI and SST were important for pilchard. Both large-scale and regional processes influenced prey taxa in variable ways. We predict that the diverse and adaptable diet of the Australian fur seal will be advantageous in a rapidly changing ecosystem.

## 1. Introduction

Climate change is accelerating variability in marine coastal environments [1], with measurable impacts being detected in both ecosystem structure and function [2]. The marine environment in southeast Australia, including Bass Strait, is one of the fastest warming marine regions in the world [2–4]. Large-scale environmental processes such as the El Niño–Southern Oscillation (ENSO), a measure of which is provided by the Southern Oscillation Index (SOI) and the Southern Annular Mode (SAM) are intensifying. SAM is driving stronger westerly winds around Antarctica, and this has flow-on effects for the middle latitudes including southern Australia [5]. To understand how climate change will affect ecosystems, we need to understand how individual species are responding to changing environmental processes [6,7].

By systematically monitoring the diet of high-trophic-level marine predators over a long time series, we can detect changes in prey availability, and by inference, ecosystem health [8–11]. Monitoring diet may provide an indication of the availability of prey within an animal's foraging range, albeit moderated by prey preferences [12] and the foraging ability of the predator [13]. The Australian fur seal (*Arctocephalus pusillus doriferus*) is a high-trophic-level marine predator that is constrained to the continental shelf and forages predominately on the shelf water of Bass Strait [14,15]. By inference, monitoring the diet of this known generalist and opportunistic predator over time, we can gain an indication of prey available to that predator within an ecosystem and infer how species assemblages have changed through time. In this study we aim to: (i) quantify temporal changes in the diet of Australian fur seals in Bass Strait, and so how local species assemblages have changed over time, and (ii) investigate if these changes correlate with key environmental variables.

Bass Strait is a shallow 50–70 m deep marine basin located between Victoria and Tasmania in southeast Australia (Cirano and Middleton [16]), and is home to 56% of the breeding population of Australian fur seals [17]. Since the cessation of commercial harvesting in 1923 and listing as a nationally protected species in 1975 [18], the population of Australian fur seals at Seal Rocks (Victoria), one of the larger Australian fur seal colonies, has made a strong recovery [19].

A cost-effective and non-invasive method commonly used in long-term diet monitoring programmes for seals is the hard part analysis of scats [20–22]. Hard part analysis is particularly applicable to fur seals as they forage in the marine environment and return to terrestrial areas to breed and rest [14,21]. This method involves the collection of fresh scat and regurgitate to recover and identify undigested prey remains within samples [21]. For fur seals, diagnostic remains include fish ear bones (otoliths) and cephalopod beaks that are unique to species [23,24]. Although different prey remains to travel through the digestive tract at different rates, scats generally represent prey consumed in the last 24–48 h [21,25]. Scats and regurgitates for diet analysis have been collected at Seal Rocks since 1997, with the aim of understanding the dynamics of Australian fur seal prey in the Bass Strait marine ecosystem.

There are many methods for the analysis of prey from mammal scats, including frequency of occurrence (FO). Frequency of occurrence shows the relative importance of the prey in the diet. However, FO is limited to detecting prey consumed from hard parts remaining in the scat and may underestimate the abundance of prey whose otoliths become severely or completely digested or prey that are not consumed in full. Biomass reconstruction is also limited as there can be uncertainty around the age class of otoliths arising from erosion as they pass through the digestive tract [20,25,26]. Despite these limitations, FO provides a consistent method for examining relative changes in the occurrence of detectable prey over time [20,27]. In this study, we focused on FO to compare linear models previously applied to the diet of this population of Australian fur seals (1997–2006) [21] and perform updated modelling that overlays this time series (1997–2014) with concurrent environmental variables. Climate anomalies have been linked to maternal provisioning and prey availability in Antarctic fur seals (*Arctocephalus gazella*) [12] and large-scale environmental changes have been shown to influence foraging success and breeding parameters of Australian fur seals, with the assumption that the environmental conditions are affecting the prey availability [28–31]. Here we investigate long-term environmental variability and its relationship with prey species consumed by Australian fur seals in Bass Strait, southeast Australia.

To interpret long-term change, it is first necessary to understand seasonal patterns within Bass Strait in order to detect any unusual conditions that may influence prey availability to Australian fur seals. Traditionally, the main current path in Bass Strait is eastward. During the austral winter–spring, prevailing westerly winds and low-pressure systems drive the eastward flow of the South Australian Current (SAC), and it flushes through, bringing sub-Antarctic surface water into Bass Strait (Sandery & Kämpf [29]). In summer the winds reverse to southeasterlies, the SAC weakens, and old water circulates in Bass Strait warming sea surface temperatures [29]. In late summer Australia's southern coastal shelves are cooled by a large seasonal coastal upwelling system that combines the Bonney Upwelling, Kangaroo Island and Eyre Peninsula Upwelling and the West Tasmanian Upwelling [30]. The regular southeasterly coastal winds and high-pressure systems of the summer (December–April) drive upwelling of nutrient-rich Antarctic waters along a total spatial extension of approximately 1500 km from Portland in Victoria to the tip of Eyre Peninsula in South Australia and along the western Tasmanian shelf [30,31]. Although enhancing upwelling, strong easterly winds may also prevent upwelled water at the Bonney Upwelling from entering Bass Strait by weakening the SAC and strengthening the East Australian Current (EAC), effectively bringing warmer nutrient-poor water into Bass Strait from the east and preventing flushing [29,31].

The large-scale environmental processes influencing ocean flow and consequent productivity in the Bass Strait marine ecosystem are the Southern Annular Mode (SAM) and the Southern Oscillation Index (SOI) [31,32]. The SAM measures variation in westerly winds oscillating around Antarctica; when SAM is positive the westerly winds contract towards the pole, creating a strong pressure gradient with strong westerlies over the pole (approx. 55° S) and weaker westerlies over southern Australia (approx. 35° S); this persists in all seasons but is strongest in summer [33]. When the SAM is negative, southern Australia experiences low-pressure weather systems and stronger westerly winds that result in cooler sea surface temperatures (SSTs), this relationship is stronger in winter and contributes to the upwelling of nutrient rich water from the Bonney Upwelling region into Bass Strait [30,33]. Climate models predict that an upward trend in SAM, possibly in response to global warming, will weaken westerly winds of southern Australia and strengthen the EAC resulting in faster warming and greater incursions into eastern Bass Strait [34]. Water from the EAC is nutrient-poor and warmer than Bass Strait water and therefore may influence productivity declines in Bass Strait.

The SOI is a measure of the sea-level pressure difference in the Pacific Ocean between Tahiti and Darwin [35]. In Australia, El Niño (La Niña) events are identified by sustained negative (positive) SOI values that are associated with weaker (stronger) Pacific trade winds, reduced (increased) rainfall in the north and east, and cooler (warmer) SSTs. Like SAM, the SOI correlates with major changes in ocean current strengths and weather patterns and may affect the productivity in Bass Strait [36] and therefore the foraging efficiency of, or prey availability to, Australian fur seals. Changes in SOI have impacted pinniped species globally [37,38]. For Australian fur seals, SOI has been linked to foraging behaviour [37], foraging effort [28] and pup production [38], suggesting influences on the availability of prey.

Wind strength and punctual events of strong winds (e.g. storms) have also been shown to influence the foraging ability and reproductive success of predators in the region [39]. As well as affecting water exchange rates in Bass Strait, wind strength may disrupt thermoclines and change prey distribution with flow-on impacts for predators including Australian fur seals. Thermoclines, where water temperatures transition from warmer water above to cooler water below, were first documented in Bass Strait in 1983 [40] and are seasonally present in summer and autumn with prevailing southeasterly winds [41]. Thermoclines have been shown to positively influence penguin foraging by concentrating prey along the thermocline edge [42]. Other pinniped species have been shown to use thermoclines while foraging [43,44], and increased sub-thermocline water temperature and the El Niño index have been negatively linked to pup production in Galapagos fur seals (*Arctocephalus galapagoensis*) and sea lions (*Zalophus wollebaeki*) [45].

In southeast Australia, sea temperature is warming four times faster than the global average [1]. Correlations between SST and changes in prey of high-trophic-level predators within the Bass Strait marine ecosystem have been identified [21,46]. Prey availability for high-trophic marine predators (Australian fur seals and penguins) has been linked with changes in SST [21] and oceanographic currents, such as the SAC and Bonney Upwelling [39]. Indeed, warmer SSTs have been predicted to negatively affect the availability of prey for marine mammals, and large-scale climate conditions may be causing Australian fur seals to work harder while foraging with less success [28,37]. Much effort has been placed on correlating environmental variables with the foraging and breeding success of Australian fur seals and the assumed response of their prey, with variable and at times conflicting results that sometimes do and do not include time lags [28,37,38,47]. However, apart from an earlier study by Kirkwood *et al*. [21] that investigated Australian fur seal prey from 1998 to 2006, little has been done to correlate environmental variables directly to Australian fur seal prey. Kirkwood *et al*. [21] detected a positive relationship between cooler SST in Bass Strait and what was at the time, a main prey species, redbait *Emmelichthys nitidus*, indicating that cooler SSTs were beneficial for the redbait and/or the ability for the seals to target them. In this study, we improve on this knowledge by exploring responses of all main prey taxa of the Australian fur seal to environmental variability.

The diet of Australian fur seals varies temporally and spatially [13,19,21,48–50] but includes fish, cephalopods, cartilaginous prey and crustaceans [51–54]. In this study, we investigate the relationship between the FO of prey taxa and environmental variables from 1997 to 2014 to determine if prey available to Australian fur seals in Bass Strait are influenced by large-scale oceanographic processes and local environmental variability. To achieve this, four potential correlates of temporal variation in the Australian fur seal diet were included in this study: SST, wind strength and direction, SAM and SOI.

## 2. Methods

### 2.1. Hard part analysis

**Figure 1.**
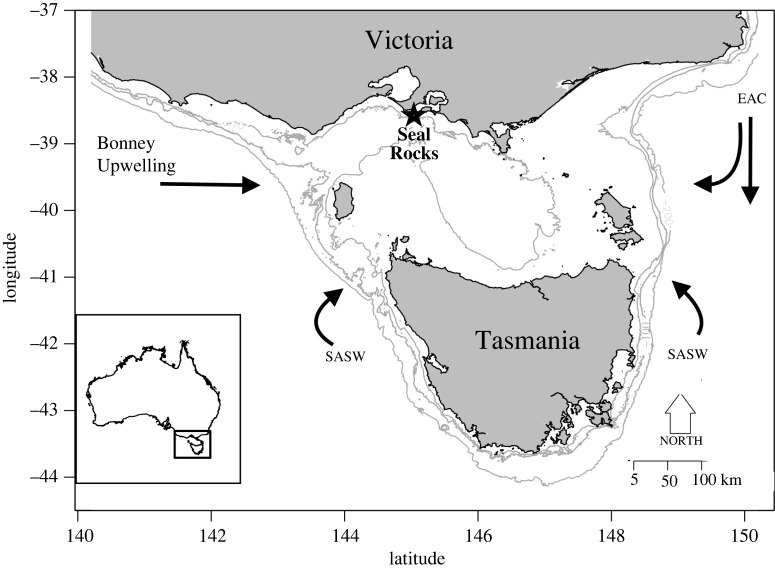
Location of Seal Rocks (star) within Bass Strait, south eastern Australia, and directions of major currents: East Australian Current (EAC); sub-Antarctic surface water (SASW) and Bonney Upwelling.

Fresh scat samples were collected approximately every two months between December 1997 and 2014 from the largest breeding colony of Australian fur seals at Seal Rocks in Victoria, Australia (38°30′ S, 145°10′ E) (figure 1). This site includes a mix of breeding and non-breeding females, juveniles, sub-adult males and breeding males throughout the year. On 105 sampling days over 17 years, 2972 samples were collected (table 1). Individual scats were stored in separate plastic bags and either processed within 48 h or frozen and sorted at a later date. Prey items were identified by an expert under a stereomicroscope (Olympus SZ-61, DP22CU) using a reference collection of hard parts including otoliths, teeth, jaws, spines, cephalopod beaks and carapace fragments, as outlined in Kirkwood *et al*. [21].

**Table 1.** The number of samples (scats) collected for hard part analysis each season from Seal Rocks, Victoria, Australia between 1998 and 2014.

	year																
season	1998	1999	2000	2001	2002	2003	2004	2005	2006	2007	2008	2009	2010	2011	2012	2013	2014
summer	44	22	17	0	19	0	0	28	11	32	60	26	63	55	0	50	39
autumn	44	41	23	11	42	21	50	27	85	30	77	58	27	30	103	62	91
winter	66	50	31	25	50	53	52	88	24	61	63	59	59	63	32	111	56
spring	17	40	28	25	24	25	52	62	56	76	32	60	60	64	51	23	46
total	171	153	99	61	135	99	154	205	176	199	232	203	209	212	186	246	232

We assumed that each sample collected represented one individual seal from a cross-section of the population. Only fresh whole scats were collected, probably representing what had passed through the digestive tract in the previous 48 h. The probability of detecting the main prey species per number of samples collected was examined *post hoc* to identify the suitable scale for modelling the environmental variables over time (sample date, season or year) and to determine target sample size for future field trips. To this end, a cumulative diversity curve using a bootstrapping function at 500 replicates in the R statistical framework v. 3.1.2 [55] was applied to the data.

The cumulative density curve (electronic supplementary material, figure S1) showed that at least 30 individual scats were required to detect changes in the main prey items of Australian fur seals (table 1). Once samples were pooled by season, sample size for most seasons over the 16 years was satisfactory, except for four years during the austral summer (2001, 2003, 2004 and 2012) when no samples were collected. In these years, access to Seal Rocks was reduced during the summer either from competing research priorities or lack of funding. Therefore, season was not investigated as an interactive term in the modelling.

### 2.2. Environmental variables

Environmental data were standardized by taking the value of a covariate and subtracting the mean of the dataset for that covariate, except where data were provided pre-standardized as anomalies. To ensure independence between the environmental data, each covariate was checked for collinearity prior to analysis and only uncorrelated covariates used for the modelling [56]. Data for model input were summarized by prey taxa, season and year, where taxa were the species or family of identified prey. The time series of all datasets for environmental covariates and prey taxa were matched from 1 January 1998 until 30 December 2014.

Sea surface temperature (SST) data—L3S Advanced Very High Resolution Radiometer (AVHRR) monthly day–night SST, 1998–2014—was obtained for the region of Bass Strait (39–41° S, 144–148° E), via the Integrated Marine Observing System (IMOS) portal (http://imos.aodn.org.au/imos/ accessed 22 April 2022). This area was chosen to cover the main foraging range of the species. Seasonal means by year were calculated from the monthly day–night values of SST and merged with the model dataset.

Wind speed and direction were obtained from a coastal weather station located 100 km from the Australian fur seal colony (Laverton Royal Australian Air Force base: GPS coordinates 37.86° S, 144.76° E; Bureau of Meteorology, Australia). The data were in a time series of eight daily measurements at 3 h intervals. Gaps in sampling effort were managed by using a subset of the daily measurements that were consistently sampled throughout the time series: taken at the hours of 0.00, 9.00 and 15.00. From this subset, mean seasonal values on an annual basis were calculated. Direction values were derived based on circular mean values from the four cardinal compass bearings north, east, south and west. Wind speed data were mean-centred and scaled to unit variance prior to modelling.

SAM anomaly data were obtained from the British Antarctic Survey, National Centre for Atmospheric Research (http://www.nerc-bas.ac.uk/icd/gjma/sam.html accessed 15 June 2015). Monthly SOI data were obtained from the NOAA Climate Prediction Centre (https://psl.noaa.gov/data/climateindices/ accessed 15 June 2022) with El Niño (La Niña) conditions defined as an extended period of negative (positive) SOI.

### 2.3. Data analysis

#### 2.3.1. Frequency of occurrence

To calculate the relative importance of each species in the diet to use in the modelling, the FO of each prey taxa was calculated at seasonal (three-month) and calendar year (annual) time scales (Hume *et al*. [49]). The FO of each prey species, *i*, was calculated as per the equation FO*_i_*= *n_i_*/(*n*–*e*), where *n_i_* is the number of samples containing species *i* in a sampling period (season or year), *n* and *e* are the total number of samples and empty samples collected in the same sampling period, respectively. ‘Empty’ samples were defined as samples with only unidentifiable and unclassifiable remains (*n* = 1458 of 2972 samples). Main prey were defined as those accounting for greater than 10% FO in any year or greater than 5% of FO across all years.

### 2.4. Modelling

We used generalized additive models (GAMs) to infer how environmental variation may be correlated with the FO of main prey in the diet of Australian fur seals per season in each year. GAMs were fitted to the FO by season for each of the eight main prey taxa identified in the diet of Australian fur seals using a binomial distribution with a logit link function. The global starting model included all covariates (SST, SAM, ENSO, wind direction, wind speed by wind direction, season and year).

The influence of SST, SAM, ENSO and wind speed were included as smooth terms (s) with a maximum of 4 knots, which was considered reasonable to avoid overfitting the model based on the amount of available data. Wind direction was included as a factor to allow the relationship between wind speed and FO to vary by the predominant wind direction in each season. Due to few seasons with winds observed from the N (0) and W (2), we restricted our analysis to winds from the E and S. Season (summer, autumn, winter or spring) was included as an integer and a cyclic cubic regression spline (bs = ‘cc’) that accounts for any expected cyclical pattern. Year was included as a random effect smooth term (bs = ‘re’) to account for sampling and other unknown sources of variation among years [57]. Interactions between covariates and season were not investigated due to inconsistent and relatively small sample sizes collected over time (table 1).

All analyses were completed using the R statistical framework v. 4.1.3 [55] with models fitted to the data using the ‘mgcv’ package v. 1.8–40 [58]. Model selection was conducted automatically when fitting the global GAM for each species by adding an additional penalty term to each smooth term, allowing penalization to zero and selection out of the model [59]. This is achieved in the ‘mgcv’ package by using the argument select = TRUE in gam(). Any terms from the global model with estimated degrees of freedom less than 1 were subsequently dropped from the final model, as were any retained terms with *p*-values > 0.05. We ensured that the Year random effect was retained in all models, and in models where the smooth interaction of wind speed by direction was selected, we ensured the main effect of wind direction was retained. We found this model selection approach to be computationally efficient given our dataset and resulted in more parsimonious models compared with selection of models ranked via AICc [52]. All models were fit using restricted maximum likelihood, which was appropriate given models with differing fixed effect structures were not compared.

We examined autocorrelation of residuals from the best-supported model for the eight main prey taxa to confirm that temporal sampling effects were minimal. Individual smooth terms from the best model for each main prey taxa (*n* = 8) were plotted to show the relationship over time (see electronic supplementary material, S2 for all plots) and, due to the large number of plots, plots for the best performing models (over 75% deviance explained) are provided in the paper. To compare results from this GAM method with the previous regression analysis performed by Kirkwood *et al*. [21], a subset of the data for redbait were analysed from 1998 to 2006.

#### 2.4.1. Prey size classes

To maximize the hard part analyses, prey size classes were estimated for the four prey taxa with the highest FO and final GAM model results above approximately 60% deviance explained: pilchards *Sardinops sagax,* redbait, jack mackerel *Trachurus declivis* and red cod *Pseudphycis bachus*. Fish lengths from pristine otoliths (*n* = 2084) were measured using stereomicroscopy (Olympus SZ61, DP2-SAL). Fish lengths were calculated using regression equations from Furlani *et al*. [24] and maturity size estimates for red cod [60], jack mackerel [61], pilchard [62] and redbait [63].

## 3. Results

### 3.1. Variation in the diet of Australian fur seals

Seventy-one prey taxa were identified across the duration of the study. Fish otoliths from 63 taxa were identified and accounted for approximately 90% of the items in the diet (*n* = 10 934) and cephalopod beaks from eight taxa accounted for less than 10% of the items in the diet (*n* = 1115).

**Figure 2.**
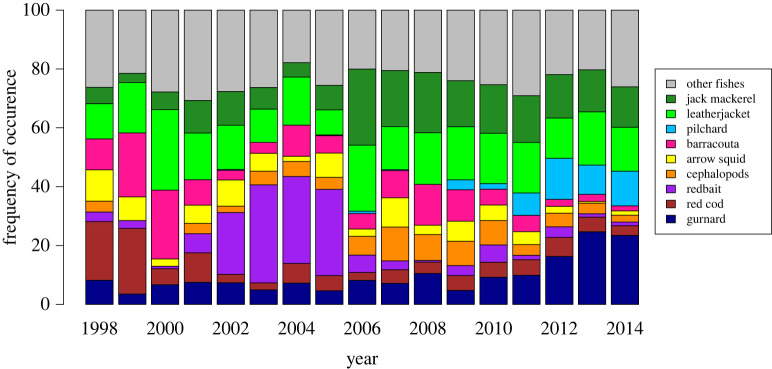
Annual frequency of occurrence (FO) of the nine main prey taxa, other fishes and cephalopods, other than arrow squid (as combined categories) identified in the diet of the Australian fur seal at Seal Rocks, Bass Strait, Australia 1998–2014.

The FO of species in the diet of Australian fur seals varied annually with eight prey items dominating. Each prey taxa accounted for greater than 10% of the FO in any single year, as did ‘other fishes’ and ‘cephalopods’ (figure 2). Overall, these eight taxa were found in more than 70% of the samples, representing the most broadly consumed prey taxa for the fur seals at Seal Rocks (i.e. the hard parts were present in more scats). These taxa satisfied our definition of main prey (greater than 10% FO in any year or greater than 5% of FO across all years) and were used for the modelling. The cumulative diversity curve showed that an asymptote was reached at 30 scat samples, with 20–32 species detected (electronic supplementary material, figure S1).

Not all of the main prey taxa were present each year. There were notable shifts in main prey items during the study (figure 2). Red cod were most prevalent in 1998 and 1999, leatherjackets in 2000 and 2001 and redbait from 2002 to 2005. From 2006, jack mackerel and leatherjacket dominated the diet. The prevalence of pilchard increased after 2009 with a maximum FO of 14% in 2012. The FO of barracouta was highest in 1999 and 2000, at 22% and 23% respectively; and gurnard FO higher from 2012 to 2014, at 16%, 25% and 23%, respectively. The most dominant cephalopod species was arrow squid spp, contributing 5% of FO over the 17 years. Other cephalopods, predominantly the cuttlefish *Sepia apama*, contributed 4.7% of the FO.

### 3.2. Prey size classes

Of the 2084 otoliths measured, 1319 (63%) were from the seven main (see above) fish prey taxa. Prey size classes were determined for four species with model results greater than 60% of the deviance explained: red cod, pilchard, jack mackerel and redbait (table 2). All pilchard were less than 84 mm indicating that only juveniles were identified in the samples. Only 3% of red cod, 0.6% of jack mackerel and 5% of redbait were of adult size.

**Table 2.** Average and range of prey sizes derived from measured otolith length (OL) for prey consumed by Australian fur seals at Seal Rocks, Victoria, Australia from 1998 to 2014. All equations are from Furlani *et al*. [24]. The symbol ^ equates to the power of. Total length is the head–tail tip; standard length measures from snout tip to end of last vertebra and fork length from snout to middle of caudal fin rays. F represents female and M represents male.

species	length	*n*	mean ± s.d. (mm)	sexual maturity (mm)	range (mm)	equation
red cod	total	127	152 ± 8	315	14–347	6.33OL^1.62
pilchard	standard	99	66 ± 3	162 (F) 172(M)	43–84	32.07OL^1.35
jack mackerel	fork	500	196 ± 4	315	84–352	16.796OL^1.3992
redbait	fork	564	141 ± 3	157 (F) 146 (M)	91–268	20.125OL^1.2238

### 3.3. Environmental correlates with frequency of occurrence

The best fitting GAM models (highest deviance explained) by main prey species are shown in table 3. All prey items varied over time (year) and all except redbait and leatherjacket included SOI as a significant correlate. Four prey taxa included SAM (redbait, pilchard, red cod and jack mackerel) and three included SST (pilchard, barracouta and red cod). Two prey taxa included wind speed and direction (redbait and leatherjacket) and the season of autumn was important for redbait. Different taxa preferred contrasting conditions. Detailed results are presented for the two best performing models (over 75% deviance explained), while complete model selection tables, assessments of time lags and residuals, are provided in the electronic supplementary material (see supplementary materials 2 fit_gams_to_FO_data.final.html). The ACF plots of the models for each main prey taxa confirmed that there is minimal autocorrelation in the data (plots provided in electronic supplementary material, figure S3).

**Table 3.** Generalized additive model results of best supported models to explain the variability in the frequency of occurrence (FO) of the eight main prey taxa of Australian fur seals at Seal Rocks, Victoria, Australia between 1998 and 2014 (*n* = 59 seasons). Models are listed by highest deviance explained and an asterisk (*) represents significant covariates (at 0.05 level) in the final model. SOI = Southern Oscillation index, SAM = Southern Annular Mode, SST = sea surface temperature. Preferred conditions of the variable are in brackets: − for negative, N for neutral, + for positive, and A for autumn. Model selection results for the eight main prey taxa are provided in the electronic supplementary material.

species	wind speed by compass direction (compared with north)			wind speed	SOI	SAM	SST	season	adjusted *r*^2^	deviance explained
	east	south	west							
redbait	* (N)	* (+)				* (+)		* (A)	0.748	82.8
pilchard					* (−,N)	* (+)	* (+)		0.799	79.3
barracouta					* (+)	* (N)	* (−)		0.481	66.0
red cod					* (−,+)		* (−)		0.546	62.4
jack mackerel					* (−,N)				0.490	59.3
gurnard					* (−,N)	* (+)			0.311	48.6
leatherjacket	*(+)	*(−,N)							0.226	38.6
arrow squid					* (+)				0.0283	12.9

**Figure 3.**
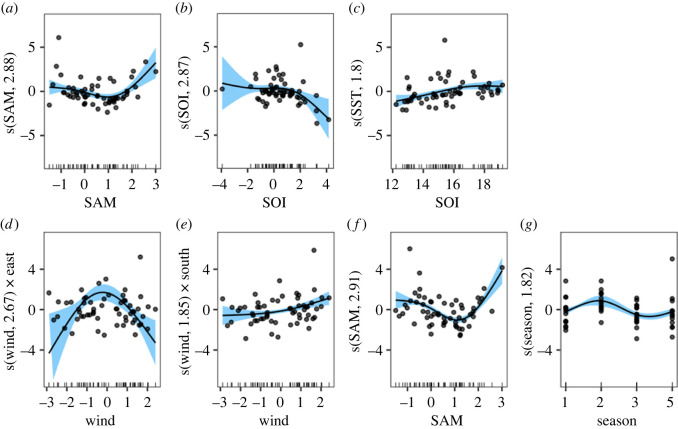
Generalized additive model results showing significant oceanographic correlations with pilchards (*a–c*) and redbait (*d–g*) as the two best performing models of the main prey of Australian fur seals with greater than 75% deviance explained. Variables include Southern Annular Mode (SAM), Southern Oscillation Index (SOI), sea surface temperature (SST) and wind represents speed and direction and seasons of the year (season) shows 1 to 4 as the Austral summer to spring.

Two main prey taxa produced especially strong correlations with explained deviances over 75%: redbait and pilchard (figure 3). For redbait, the FO in the diet was influenced both regionally and locally. Neutral and stronger winds from the east and stronger winds from the south, positive SAM conditions and the Austral season of autumn (March–May) were conducive to higher FO in the diet. For pilchards, regional influences were important with positive SAM indices, negative SOI (El Niño) and, locally, warmer SST correlated with higher FO in the diet. Both species had similar responses to SAM (figure 3).

A reanalysis of our 16 years dataset, subset to match the study period of Kirkwood *et al*. [21] showed that SST correlated with redbait, but in the opposite direction—with redbait preferring warmer temperatures in this study (electronic supplementary material, S4).

## 4. Discussion

We found temporal variation in prey taxa in the diet of Australian fur seals at Seal Rocks, Bass Strait over a 17-year period from 1998 to 2014, and in so doing extended an earlier 9-year time series [21]. This prolonged time series revealed that with extended time we had much higher prey diversity, 71 prey items overall compared with 49 from the first 9 years [21]. Our results are comparable in diversity to a recent study using DNA meta-barcoding of the prey of Australian fur seals in New South Wales, Australia that found 76 prey taxa [52].

We found significant correlations between environmental variables and prey consumed by Australian fur seals. Large-scale environmental processes, indicated by the ENSO and SAM, are known to drive physical and biological processes within marine ecosystems [64–66]. Therefore, we expected these processes to influence prey assemblages within Bass Strait. This dataset spanned multiple ENSO events and fluctuations in the SAM. Environmental variability influenced prey FO at regional (ENSO, and SAM) and local scales (SST and wind) (table 3). For Australian fur seals, foraging behaviour, foraging effort and foraging sucess have been linked to regional-, large- and local-scale environmental variability [28,37,38,47] and a single prey taxa, redbait, to SST [67]. In this study, the most influential variables were SOI, SAM and SST.

Two prey species stood out in the models: pilchard and redbait, both with greater than 75% deviance explained (table 3, figure 3). Pilchard was identified in the diet during negative (El Niño) and neutral SOI conditions, warmer SST in the foraging region and positive SAM conditions. For redbait, neutral easterly winds, stronger wind from the south, the season of autumn and positive SAM conditions resulted in higher occurrence in the diet. Negative SOI (El Niño) is expected to produce warmer SSTs, albeit regional signals may not be reflected in local conditions. Overall, positive SAM conditions can also lead to warmer SST. Different species respond in different ways to environmental variables and regional and local signals may not always be directly linked. This complexity can make it difficult to determine dominant patterns in ecosystems when using data for multiple species.

The FO of six prey taxa correlated SOI as a measure of ENSO, but with varying responses: jack mackerel, gurnard and pilchard were found under negative (El Niño) and neutral SOI; barracouta and arrow squid were found under positive SOI conditions (La Niña); while red cod FO was lowest during neutral SOI conditions. The breeding biology of jack mackerel and the predominantly neutral ENSO conditions in our study may explain their persistence as a main prey species throughout our time series.

In Australian fur seals from Kanowna Island in Bass Strait (120 km southeast of Seal Rocks), growth of teeth dentine layers, an index of body growth, was linked to environmental parameters, with body condition positively linked to the SOI (Knox *et al*. [67]). In La Niña years, seals grew more, indicating higher nutrient prey was available. Two La Niña events occurred during this study, in 1998–2000 and 2010–2011, and during these events the FOs for redbait, pilchard and gurnard were higher. Redbait FO was also greater in autumn in 2010–2011, perhaps also responding to La Niña conditions.

La Niña events occur across the entire Western Pacific region, and have a direct impact on the East Australian Current (EAC) during the Austral summer and autumn months. At this time, the EAC strengthens down the east coast of Australia, partially blocking water flow through Bass Strait and thereby increasing the residence time of water in the strait [46]. With the reduced water passage rate and low wind speeds, thermoclines can develop during summer and autumn [42]. Thermoclines can influence the distribution of prey in the water column as prey concentrate within their preferred temperature regime, focusing the foraging behaviour of marine predators, as shown by little penguins [68] and northern fur seals (*Callorhinus ursinus*) [42,43]. Interestingly, the FO of pilchard, also important prey of little penguins, was higher for Australian fur seals in El Niño and neutral SOI conditions. Their recent (2009) inclusion in the diet of the fur seals may be more related to the pilchard recovery from a herpes virus that decimated the population during the 1990s [69–71] than ENSO, particularly given El Niño conditions occurred only once after this time. Their presence in the fur seal diet after 2008 coincides with the identified population recovery in catch records of the Small Pelagic Fishery (SPF) [71].

Pilchard and redbait preferred positive SAM, whereas gurnard FO correlated to both positive and neutral SAM conditions. Positive SAM conditions in southern Australia result in weaker westerly winds, allowing the EAC to enter Bass Strait, SST increase and thermoclines to develop. This possibly improves foraging conditions for baitfish, such as redbait and pilchard, that may aggregate on the thermoclines. Gurnard, bottom-feeding carnivores [72], also preferred positive SAM conditions; however, it is uncertain why. Aligning with the results for SAM, local SST correlated positively with the FO of pilchard; but was negatively correlated with the FO of red cod and barracouta.

Kirkwood *et al*. [21] found redbait preferred cooler SST; however, we found no correlation with SST in the full dataset to 2014 and the opposite relationship in the subset data to 2006, where higher FO was correlated to higher SST. This could be due to different SST data source and/or that Kirkwood *et al*. [21] used monthly FO and we used seasonally summarized FO for the modelling. It could also be caused by the larger area of ocean used to obtain the SST data in this study.

Wind speed by compass direction was influential for two prey taxa, with redbait FO correlating to neutral easterly winds and strong southerly winds, while leatherjacket FO correlated to strong easterly and weak southerly winds. Such mixed responses are difficult to interpret, but easterly winds may bring nutrient-rich sub-Antarctic water into eastern Bass Strait [29], and although little is known of their migrations [73], strong southerly winds could influence the movement of redbait through central Bass Strait. Leatherjackets were consistent in the diet of the Australian fur seal with low deviance explained by the model suggesting it is a reliable food source despite the variable environment. When considering all the modelling results, it is clear that preferred oceanic and environmental conditions vary by prey taxa, demonstrating the need for Australian fur seal to be opportunistic and change their diet as needed.

Overall, oceanographic variability can influence prey availability and hence the reproductive and foraging success of Australian fur seals. However, these complex relationships sometimes contrast and can be difficult to interpret. The use of broad indices (e.g. pup production and foraging behaviours) when there is high individual variability in this species [28,74] may hinder clarity. For example, over a 19-year dataset, Joly *et al*. [75] found that for little penguins from Phillip Island, near to Seal Rocks and also in Bass Strait, short-term physical impediments to finding prey (waves and currents) were more important to little penguin foraging and breeding success than degrees of water stratification, ENSO or the Antarctic Oscillation. Unlike little penguins, Australian fur seals cannot adjust their breeding phenology, with an annual cycle and pups born in the Australian spring–summer then being dependent for 8–10 months, so being able to prey switch is critical to ensure survival and reproductive success.

Based on otolith measurements, the redbait, red cod and jack mackerel consumed by fur seals at Seal Rocks were predominantly juvenile (greater than 95%). Juveniles of these taxa can occur in separate areas to adults. For jack mackerel, smaller fish are found inshore and larger fish offshore [73]. Redbait may also school by size with larger fish in deeper water closer to the seafloor (Markina & Boldyrev in [73]). Similarly, juvenile pilchards may be found in shallow embayments and semi-protected waters [73]. Perhaps the seals overlap with more juvenile fish during foraging trips. Alternatively, there is active selection for the smaller bodied fish, which may be easier to catch or quicker to process [76].

The main external pressures potentially influencing small pelagic fishes are changes in environmental conditions (probably climate related) and changes in fishing pressure [77]. Using ecosystem models, it is appears that the heavily fished eastern Bass Strait (EBS) has top-down controlling elements [77]. Top-down systems are those where lower trophic levels are controlled by higher predators including commercial fishing and are more sensitive to predation and stressors, bottom-up systems are those where abundances of large predators are controlled by the availability of lower trophic groups [77]. In the EBS, Australian fur seals were nearly always top-down controlling their predator/prey interactions [77]. Simulated loss of jack mackerel and redbait in eastern Bass Strait did not cause broad change in the ecosystem but did have a small negative effect on seals in the models [77]. Based on these classifications, stresses such as climate change were considered very likely to have big impacts in the region. In this diet study, we show that the Australian fur seal diet at Seal Rocks has changed, with main prey species varying temporally and oceanographic variability influencing the FO of prey in the diet, except for leatherjackets that were a staple. We anticipate further change in this region that will probably have positive effects on some taxa and negative effects on others.

The recovery of the population of Australian fur seals breeding at Seal Rocks may have influenced FO of prey in their diet. As the population of a top predator grows, prey choice may be dominated more by intraspecific competition than prey abundance [78]; while the population of Australian fur seals at Seal Rocks increased up to 2007, we did not have annual population data to test this [79]. Other factors that were not investigated may also have influenced variation in the diet of Australian fur seals. For example, Chlorophyll *a*, typically used to represent primary productivity in models, commercial fisheries and other ecosystem effects (i.e. competition with other predators such as little penguins) may have influenced prey abundance and therefore the presence of prey in the diet of Australian fur seals.

Prey such as jack mackerel, redbait and pilchards are commercially valuable species, caught in the Commonwealth Small Pelagic Fishery [80]. Historically, commercial fishing in the form of trawling was more active in this region but has since been reduced by a buyback scheme that commenced in 2005/2006 [81]. This scheme included the purchasing of 34% of fishing licences by the Commonwealth Government, therefore reducing fishing pressure in the region [81]. The introduction of quota restrictions based on ecosystem modelling has further reduced fishing pressure in Bass Strait over the course of this study [80]. Kirkwood *et al*. [21] did not find any relationship between prey assemblages and catch per unit effort of fisheries operating within 200 km of Seal Rocks. However, a reduction in trawling activity coincided with notable shifts including the decreased frequency of occurrence of redbait and increased FO in jack mackerel in the Australian fur seal's diet during 2006 (figure 2). These fisheries are considered sustainable with small annual catches in eastern Australia [73]. Future research could include updating regional ecosystem models [77] to include an analysis of the diet of the Australian fur seals and current fisheries take.

This study is the longest time series of Australian fur seal diet, not only identifying correlations between prey FO and environmental variables but also consolidating our understanding of the adaptability of this generalist predator. FO is a measure that identifies changes in the relative importance of prey in the diet, demonstrating that prey of Australian fur seals has changed over time, inferring changes in prey species assemblages in Bass Strait. This study further confirms that large-scale environmental processes do influence ecological change and predator–prey dynamics in the Bass Strait ecosystem. These correlations suggest regional and local processes may influence individual prey taxa differently and highlight the benefits of being an opportunistic and diverse predator. Climate change impacts ecosystems in multiple ways including upwelling frequency and thermocline stability, ocean productivity and larval recruitment [77]. We predict that the adaptable foraging strategy of the Australian fur seal will be advantageous in a rapidly changing ecosystem, particularly given this species and marine mammals in general are predicted to be negatively affected by climate change.

## Data Availability

The data used in this study are available on Figshare at https://doi.org/10.6084/m9.figshare.6795164.v1. The data are provided in electronic supplementary material [82].

## References

[1] IPCC. 2014 Climate Change 2014: *Synthesis Report. Contribution of Working Groups I, II and III to the Fifth Assessment Report of the Intergovernmental Panel on Climate Change* (eds Core Writing Team, RK Pachauri, LA Meyer), 151pp. Geneva, Switerland: IPCC. In IPCC AR5 Synthesis Report website. See https://www.ipcc.ch/report/ar5/syr/.

[2] Last PR, White WT, Gledhill DC, Hobday AJ, Brown R, Edgar GJ, Pecl G. 2011 Long-term shifts in abundance and distribution of a temperate fish fauna: a response to climate change and fishing practices. Glob. Ecol. Biogeogr. **20**, 58-72. (10.1111/j.1466-8238.2010.00575.x)

[3] Chambers LE, Patterson T, Hobday AJ, Arnould JPY, Tuck GN, Wilcox C, Dann P. 2015 Determining trends and environmental drivers from long-term marine mammal and seabird data: examples from Southern Australia. Reg. Environ. Change **15**, 197-209. (10.1007/s10113-014-0634-8)

[4] Hobday AJ, Pecl GT. 2014 Identification of global marine hotspots: sentinels for change and vanguards for adaptation action. Rev. Fish Biol. Fish. **24**, 415-425. (10.1007/s11160-013-9326-6)

[5] Marshall GJ. 2003 Trends in the Southern annular mode from observations and reanalyses. J. Clim. **16**, 4134-4143. (10.1175/1520-0442(2003)016<4134:TITSAM>2.0.CO;2)

[6] Otto SA, Kornilovs G, Llope M, Möllmann C. 2014 Interactions among density, climate, and food web effects determine long-term life cycle dynamics of a key copepod. Mar. Ecol. Prog. Ser. **498**, 73-84. (10.3354/meps10613)

[7] Schmidt AE, Dybala KE, Botsford LW, Eadie JM, Bradley RW, Jahncke J. 2015 Shifting effects of ocean conditions on survival and breeding probability of a long-lived seabird. PLoS ONE **10**, e0132372. (10.1371/journal.pone.0132372) 26168050 PMC4500586

[8] Boyd IL, Murray AWA. 2001 Monitoring a marine ecosystem using responses of upper trophic level predators. J. Anim. Ecol. **70**, 747-760. (10.1046/j.0021-8790.2001.00534.x)

[9] Hindell M, Bradshaw C, Harcourt R, Guinet C. 2003 17 Ecosystem monitoring: are seals a potential tool for monitoring change in marine systems? Books Online **2006**, 330-343.

[10] Hobday AJ, Arrizabalaga H, Evans K, Nicol S, Young JW, Weng KC. 2015 Impacts of climate change on marine top predators: advances and future challenges. Deep Sea Res. Part II Top Stud. Oceanogr. **113**, 1-8. (10.1016/j.dsr2.2015.01.013)

[11] Trathan PN, Forcada J, Murphy EJ. 2007 Environmental forcing and Southern Ocean marine predator populations: effects of climate change and variability. Phil. Trans. R. Soc. B **362**, 2351-2365. (10.1098/rstb.2006.1953) 17553770 PMC2443178

[12] Lea MA, Guinet C, Cherel Y, Duhamel G, Dubroca L, Pruvost P, Hindell M. 2006 Impacts of climatic anomalies on provisioning strategies of a Southern Ocean predator. Mar. Ecol. Prog. Ser. **310**, 77-94. (10.3354/meps310077)

[13] Page B, McKenzie J, Goldsworthy SD. 2005 Dietary resource partitioning among sympatric New Zealand and Australian fur seals. Mar. Ecol. Prog. Ser. **293**, 283-302. (10.3354/meps293283)

[14] Arnould JPY, Hindell MA. 2001 Dive behaviour, foraging locations, and maternal-attendance patterns of Australian fur seals (*Arctocephalus pusillus doriferus*). Can. J. Zool. **79**, 35-48. (10.1139/z00-178)

[15] Salton M, Kirkwood R, Slip D, Harcourt R. 2019 Mechanisms for sex-based segregation in foraging behaviour by a polygynous marine carnivore. Mar. Ecol. Prog. Ser. **624**, 213-226. (10.3354/meps13036)

[16] Cirano M, Middleton JF. 2004 Aspects of the mean wintertime circulation along Australia’s southern shelves: numerical studies. J. Phys. Oceanogr. **34**, 668-684. (10.1175/2509.1)

[17] McIntosh R, Kirkman SP, Sutherland D, Mitchell T, Arnould JPY, Kirkwood R. 2018 Understanding meta-population trends of the Australian fur seal, with insights for adaptive monitoring. PLoS ONE **13**, e0200253. (10.1371/journal.pone.0200253) 30183713 PMC6124711

[18] Victorian State Government. 1975 Wildlife Act. Victoria, Australia: Victorian State Government.

[19] Kirkwood R, Goldsworthy S. 2013 Fur seals and sea lions. Melbourne, Australia: CSIRO.

[20] Bowen WD, Iverson SJ. 2013 Methods of estimating diets: a review of validation experiments and source of bias and uncertainty. Mar. Mammal Sci. **29**, 719-754. (10.1111/j.1748-7692.2012.00604.x)

[21] Kirkwood R, Hume F, Hindell M. 2008 Sea temperature variations mediate annual changes in the diet of Australian fur seals in Bass Strait. Mar. Ecol. Prog. Ser. **369**, 297-309. (10.3354/meps07633)

[22] Tollit DJ, Wong MA, Trites AW. 2015 Diet composition of Steller sea lions (*Eumetopias jubatus*) in Frederick Sound, southeast Alaska: a comparison of quantification methods using scats to describe temporal and spatial variabilities. Can. J. Zool. **93**, 361-376. (10.1139/cjz-2014-0292)

[23] Lu CC, Ickeringill R. 2002 Cephalopod beak identification and biomass estimation techniques: tools for dietary studies of southern Australian finfishes. Mus. Vic. Sci. Rep. **6**, 1-65. (10.24199/j.mvsr.2002.06)

[24] Furlani D, Gales R, Pemberton D. 2007 Otoliths of common temperate Australian fish: a photographic guide. Melbourne, Australia: CSIRO.

[25] Fea Nl, Harcourt R. 1997 Assessing the use of faecal and regurgitate analysis as a means of determining the diet of New Zealand fur seals. Mar. Mammal. Res. South Hemisphere **1**, 143-150.

[26] Klare U, Kamler JF, Macdonald DW. 2011 A comparison and critique of different scat-analysis methods for determining carnivore diet. Mammal. Rev. **41**, 294-312. (10.1111/j.1365-2907.2011.00183.x)

[27] Tollit DJ, Steward MJ, Thompson PM, Pierce GJ, Santos MB, Hughes S. 1997 Species and size differences in the digestion of otoliths and beaks: implications for estimates of pinniped diet composition. Can. J. Fish. Aquat. Sci. **54**, 105-119. (10.1139/f96-264)

[28] Speakman CN, Hoskins AJ, Hindell M, Costa DP, Hartog J, Hobday AJ, Arnould JP. 2020 Environmental influences on foraging effort, success and efficiency in female Australian fur seals. Sci. Rep. **10**, 1-16. (10.1038/s41598-020-73579-y) 33077806 PMC7572486

[29] Sandery PA, Kämpf J. 2007 Transport timescales for identifying seasonal variation in Bass Strait, south-eastern Australia. Estuar. Coast. Shelf Sci. **74**, 684-696. (10.1016/j.ecss.2007.05.011)

[30] Kämpf J, Doubell M, Griffin D, Matthews RL, Ward T. 2004 Evidence of a large seasonal coastal upwelling system along the southern shelf of Australia. Geophys. Res. Lett. **31**, 1-4. (10.1029/2003gl019221)

[31] Middleton JF, Bye JA. 2007 A review of the shelf-slope circulation along Australia's southern shelves: Cape Leeuwin to Portland. Prog. Oceanogr. **75**, 1-41. (10.1016/j.pocean.2007.07.001)

[32] Harris G, Nilsson C, Clementson L, Thomas D. 1987 The water masses of the east coast of Tasmania: seasonal and interannual variability and the influence on phytoplankton biomass and productivity. Mar. Freshw. Res. **38**, 569-590. (10.1071/MF9870569)

[33] Lovenduski NS. 2005 Impact of the southern annular mode on Southern Ocean circulation and biology. Geophys. Res. Lett., 1-4. (10.1029/2005GL022727)

[34] Cai W, Shi G, Cowan T, Bi D, Ribbe J. 2005 The response of the Southern Annular Mode, the East Australian Current, and the southern mid-latitude ocean circulation to global warming. Geophys. Res. Lett. **32**. (10.1029/2005GL024701)

[35] Salisbury J, Wimbush M. 2002 Using modern time series analysis techniques to predict ENSO events from the SOI time series. Nonlinear Process Geophys. **9**, 341-345. (10.5194/npg-9-341-2002)

[36] Jordan A, Pullen G, Marshall J, Williams H. 1995 Temporal and spatial patterns of spawning in jack mackerel, *Trachurus declivis* (Pisces: Carangidae), during 1988–91 in eastern Tasmanian waters. Mar. Freshw. Res. **46**, 831-842. (10.1071/MF9950831)

[37] Hoskins AJ, Arnould JP. 2014 Relationship between long-term environmental fluctuations and diving effort of female Australian fur seals. Mar. Ecol. Prog. Ser. **511**, 285-295. (10.3354/meps10935)

[38] Geeson J, Hobday A, Speakman CN, Arnould JPY. 2022 Environmental influences on breeding biology and pup production in Australian fur seals. R. Soc. Open Sci. **9**, 211399. (10.1098/rsos.211399) 35425634 PMC9006029

[39] Saraux C, Chiaradia A, Salton M, Dann P, Viblanc VA. 2016 Negative effects of wind speed on individual foraging performance and breeding success in little penguins. Ecol. Monogr. **86**, 61-77. (10.1890/14-2124.1)

[40] Jones I, Padman L. 1983 Semidiurnal and internal tides in eastern Bass Strait. Aust. J. Mar. Freshw. Res. **34**, 159-171. (10.1071/MF9830159)

[41] Jones I. 1980 Tidal and wind-driven currents in Bass Strait. Aust. J. Mar. Freshw. Res. **31**, 109-117. (10.1071/MF9800109)

[42] Pelletier L, Kato A, Chiaradia A, Ropert-Coudert Y. 2012 Can thermoclines be a cue to prey distribution for marine top predators? A case study with little penguins. PLoS ONE **7**, e31768. (10.1371/journal.pone.0031768) 22536314 PMC3335045

[43] Nordstrom CA, Battaile B, Cotte C, Trites AW. 2013 Foraging habitats of lactating northern fur seals are structured by thermocline depths and submesoscale fronts in the eastern Bering Sea. Deep Sea Res. Part II Top Stud. Oceanogr. **88**, 78-96. (10.1016/j.dsr2.2012.07.010)

[44] Kuhn CE. 2011 The influence of subsurface thermal structure on the diving behavior of northern fur seals (*Callorhinus ursinus*) during the breeding season. Mar. Biol. **158**, 649-663. (10.1007/s00227-010-1589-z)

[45] Páez-Rosas D, Torres J, Espinoza E, Marchetti A, Seim H, Riofrío-Lazo M. 2021 Declines and recovery in endangered Galapagos pinnipeds during the El Niño event. Sci. Rep. **11**, 1-15. (10.1038/s41598-021-88350-0) 33888850 PMC8075323

[46] Gibbs CF. 1992 Oceanography of Bass Strait: implications for the food supply of little penguins *Eudyptula minor*. Emu **91**, 395-401. (10.1071/MU9910395)

[47] Gibbens JR, Arnould JPY. 2009 Interannual variation in pup production and the timing of breeding in benthic foraging Australian fur seals. Mar. Mammal Sci. **25**, 573-587. (10.1111/j.1748-7692.2008.00270.x)

[48] Arnould JPY, Cherel Y, Gibbens J, White JG, Littnan CL. 2011 Stable isotopes reveal inter-annual and inter-individual variation in the diet of female Australian fur seals. Mar. Ecol. Prog. Ser. **422**, 291-302. (10.3354/meps08933)

[49] Hume F, Hindell M, Pemberton D, Gales R. 2004 Spatial and temporal variation in the diet of a high trophic level predator, the Australian fur seal (*Arctocephalus pusillus doriferus*). Mar. Biol. **144**, 407-415. (10.1007/s00227-003-1219-0)

[50] Kernaléguen L, Cherel Y, Knox TC, Baylis A, Arnould JPY. 2015 Sexual niche segregation and gender-specific individual specialisation in a highly dimorphic marine mammal. PLoS ONE **10**, e0133018. (10.1371/journal.pone.0133018) 26244371 PMC4526469

[51] Deagle BE, Kirkwood R, Jarman SN. 2009 Analysis of Australian fur seal diet by pyrosequencing prey DNA in faeces. Mol. Ecol. **18**, 2022-2038. (10.1111/j.1365-294X.2009.04158.x) 19317847

[52] Hardy N. 2017 Assessing the trophic ecology of top predators across a recolonisation frontier using DNA metabarcoding of diets. Mar. Ecol. Prog. Ser. **573**, 237-254. (10.3354/meps12165)

[53] Kirkwood R, McIntosh R. 2021 Australian fur seal: adapting to coexist in a shared ecosystem. In Ethology and behavioral ecology of otariids and the odobenid (eds C Campahna, R Harcourt), pp. 587-620. Berlin, Germany: Springer.

[54] McNally J, Lynch DD. 1954 Notes on the food of Victorian seals. Melbourne, Australia: Victoria Fisheries and Game Department.

[55] R Core Team. 2015 A language and environment for statistical computing. Vienna, Austria: R Foundation for Statistical Computing. See https://www.R-project.org/.

[56] Zurr AF, Ieno EN, Elphick CS. 2010 A protocol for data exploration to avoid common statistical problems. Methods Ecol. Evol. **1**, 3-14. (10.1111/j.2041-210X.2009.00001.x)

[57] Wood S. 2006 Generalized additive models: an introduction with R. Boca Raton, FL: Chapman Hall/ CRC.

[58] Wood SN. 2017 Generalized additive models: an introduction with R, 2nd edition. Boca Raton, FL: Chapman and Hall/CRC.

[59] Wood SN. 2008 Fast stable direct fitting and smoothness selection for generalized additive models. J. Roy. Statist. Soc. B. **70**, 495-518.

[60] Kemp J, Jenkins GP, Swearer SE. 2012 The reproductive strategy of red cod, *Pseudophycis bachus*, a key prey species for high trophic-level predators. Fish. Res. **125**, 161-172. (10.1016/j.fishres.2012.02.021)

[61] Marshall J, Pullen G, Jordan A. 1993 Reproductive biology and sexual maturity of female jack mackerel, *Trachurus declivis (Jenyns)*, in eastern Tasmanian waters. Mar. Freshw. Res. **44**, 799-809. (10.1071/MF9930799)

[62] Fletcher WJ. 1995 Application of the otolith weight – age relationship for the pilchard, *Sardinops sagax neopilchardus*. Can. J. Fish. Aquat. Sci. **52**, 657-664. (10.1139/f95-066)

[63] Ewing GP, Lyle JM. 2009 Reproductive dynamics of redbait, *Emmelichthys nitidus* (Emmelichthyidae), from south-eastern Australia. Fish. Res. **97**, 206-215. (10.1016/j.fishres.2009.02.007)

[64] Dann P, Chambers L. 2013 Ecological effects of climate change on little penguins *Eudyptula minor* and the potential economic impact on tourism. Clim. Res. **58**, 67-79. (10.3354/cr01187)

[65] Fogt RL, Bromwich DH. 2006 Decadal variability of the ENSO teleconnection to the high-latitude South Pacific governed by coupling with the Southern Annular Mode. J. Clim. **19**, 979-997. (10.1175/JCLI3671.1)

[66] Sebille E, Sprintall J, Schwarzkopf FU, Sen Gupta A, Santoso A, England MH, Biastoch A, Böning CW. 2014 Pacific-to-Indian Ocean connectivity: Tasman leakage, Indonesian throughflow, and the role of ENSO. J. Geophys. Res. Oceans. **119**, 1365-1382. (10.1002/2013JC009525)

[67] Knox TC, Stuart-Williams H, Warneke RM, Hoskins AJ, Arnould JPY. 2014 Analysis of growth and stable isotopes in teeth of male Australian fur seals reveals interannual variation in prey resources. Mar. Mammal Sci. **30**, 763-781. (10.1111/mms.12078)

[68] Carroll G, Everrett J, Harcourt R, Slip D, Jonsen I. 2016 High sea surface temperatures driven by a strengthening current reduce foraging success by penguins. Sci. Rep. **6**, 22236. (10.1038/srep22236) 26923901 PMC4770590

[69] Ward T, Hoedt F, McLeay WF, Dimmlich WF, Kinloch M, Jackson GD, McGarvey R, Rogers PJ, Jones K. 2011 Effects of the 1995 and 1998 mass mortality events on the spawning biomass of sardine, *Sardinops sagax*, in South Australian waters. ICES J. Mar. Sci. **58**, 865-875. (10.1006/jmsc.2001.1077)

[70] Chiaradia A, Forero MG, Hobson KA, Cullen M. 2010 Changes in diet and trophic position of a top predator 10 years after a mass mortality of a key prey. ICES J. Mar. Sci. J. Cons. **67**, 1710-1720. (10.1093/icesjms/fsq067)

[71] Whittington R, Crockford M, Jordan D, Jones B. 2008 Herpesvirus that caused epizootic mortality in 1995 and 1998 in pilchard, *Sardinops sagax neopilchardus* (Steindachner), in Australia is now endemic. J. Fish Dis. **31**, 97-105. (10.1111/j.1365-2761.2007.00869.x) 18234017

[72] Park JM, Coburn E, Platell ME, Gaston TF, Taylor MD, Williamson JE. 2017 Diets and resource partitioning among three sympatric gurnards in Northeastern Tasmanian Waters, Australia. Mar. Coast. Fish. **9**, 305-319. (10.1080/19425120.2017.1320342)

[73] Grammar GL, Ward T, Durante L. 2022 *Commonwealth Small Pelagic Fishery: Fishery Assessment Report 2019–2021*. Report to the Australian Fisheries Management Authority. SARDI Publication No. F2010/000270-11. SARDI Research Report Series No. 1133. Adelaide, Australia: South Australian Research and Development Institute (Aquatic Sciences).

[74] Hoskins AJ, Schumann N, Costa DP, Arnould JPY. 2017 Foraging niche separation in sympatric temperate-latitude fur seal species. Mar. Ecol. Prog. Ser. **566**, 229-241. (10.3354/meps12024)

[75] Joly NB, Chiaradia A, Georges JY, Saraux C. 2022 Environmental effects on foraging performance in little penguins: a matter of phenology and short-term variability. MEPS **692**, 151-168. (10.3354/meps14058)

[76] Hocking DP, Salverson M, Fitzgerald EM, Evans AR. 2014 Australian fur seals (*Arctocephalus pusillus doriferus*) use raptorial biting and suction feeding when targeting prey in different foraging scenarios. PLoS ONE **9**, e112521. (10.1371/journal.pone.0112521) 25390347 PMC4229231

[77] Bulman CM, Condie SA, Neira FJ, Goldsworthy S, Fulton EA. 2011 The trophodynamics of small pelagic fishes in the southern Australian ecosystem and the implications for ecosystem modelling of southern temperate fisheries. Final report for FRDC project 2008/023. CSIRO Marine and Atmospheric Research.

[78] Kuhn CE, Baker JD, Towell RG, Ream RR. 2014 Evidence of localized resource depletion following a natural colonization event by a large marine predator. J. Anim. Ecol. **83**, 1169-1177. (10.1111/1365-2656.12202) 24450364

[79] McIntosh R, Sorrwll KJ, Thalman S, Mitchell T, Gray R, Schnagl H, Arnould JP, Dann P, Kirkwood R. 2022 Sustained reduction in numbers of Australian fur seal pups: implications for future population monitoring. PLoS ONE **17**, e0265610. (10.1371/journal.pone.0265610) 35303037 PMC8932563

[80] Ward T, Ivery AR, Earl J. 2014 *Commonwealth Small Pelagic Fishery: Fishery Assessment Report 2013*. Report to the Australian Fisheries Management Authority. South Australian Research and Development Institute (Aquatic Sciences), Adelaide. SARDI Publication No. F2010/000270-5. SARDI Research Report Series. p. 105. Report No. 788.

[81] Minnegal M, Dwyer PD. 2008 Mixed messages: buying back Australia's fishing industry. Mar. Policy **32**, 1063-1071. (10.1016/j.marpol.2008.03.005)

[82] Kliska K, McIntosh RR, Jonsen I, Hume F, Dann F, Kirkwood R, Harcourt R. 2022 Data from: Environmental correlates of temporal variation in the prey species of Australian fur seals inferred from scat analysis. Figshare. (10.6084/m9.figshare.c.6214766) PMC9532993 36249336

